# Supplementary material for: Neuronal populations across the cortex underlie discrete, categorical, and subjective representations of visual durations
Source: PLoS Biol. 2026 Mar 26;24(3):e3003704. doi: 10.1371/journal.pbio.3003704 (PMC13020800; doi:10.1371/journal.pbio.3003704)
Supplement: S1 Fig — Top: group-level t-value clusters (p < 0.001 FWE-corrected for multiple comparisons at the cluster level) are displayed color-coded (from red, t = 4, to yellow, t = 10.7) on a common (fsaverage) flattened cortical surface. Bottom: the 63 ROIs (47 in the left hemisphere and 46 in the right hemisphere) used in the analyses are shown. They encompass the functional activations displayed in the top panel and were extracted from the HCP MMP 1.0 atlas and Sereno’s topological atlas (see Methods - Regions of interest (ROIs) identification). ROI labels are displayed in white and follow the nomenclature of the atlases. ROIs are color-coded according to functional streams: green for ventral visual (VV), blue lateral visual (LV), violet for IPS, purple for inferior parietal (IP), red for motor-somatosensory (mot-som), brown for SMA, orange for premotor (PM), ochre for anterior insula (AI), yellow for inferior frontal (IF). Major sulci are displayed as thick semi-transparent white lines, with the following abbreviations: CAS = calcarine sulcus, LOS = lateral occipital sulcus, ITS = inferior temporal sulcus, STS = superior temporal sulcus, IPS = intraparietal sulcus, SF = Sylvian fissure, CS = central sulcus, IFS = inferior frontal sulcus, SFS = superior frontal sulcus, preCS = precentral sulcus. (PDF) [file pbio.3003704.s001.pdf]

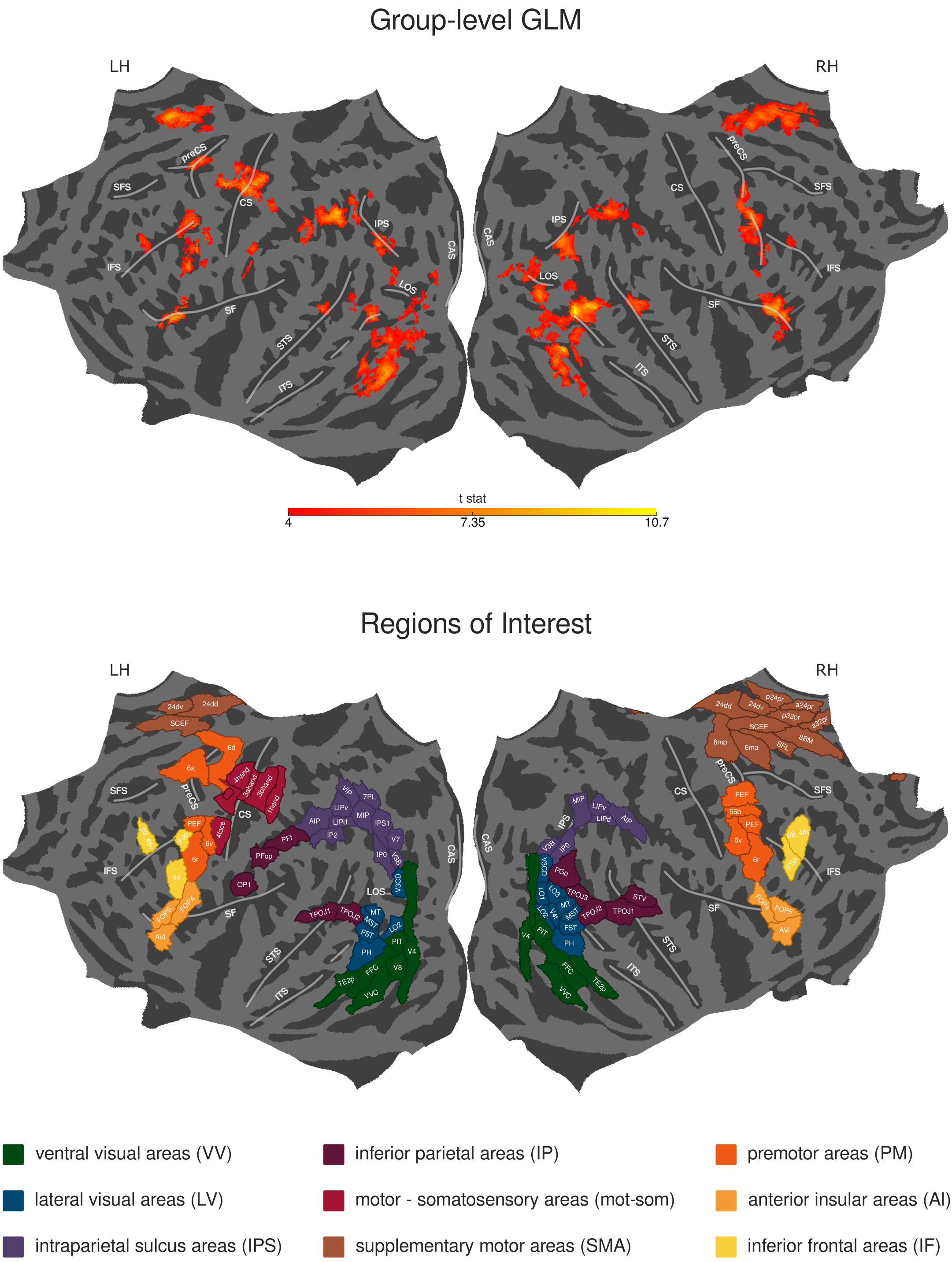
Group-level GLM
LH
RH
SFS
preCS
CS
IPS
IFS
SF
LOS
CAS
STS
ITS
t stat
4
7.35
10.7
Regions of Interest
LH
RH
24dv
24dd
SCEF
6d
6a
4hand
3ahand
3bhand
1hand
PEF
6v
6r
4face
IFJp
p9-46v
44
FOP4
FOP5
AVI
OP1
PFop
PFt
AIP
LIPv
LIPd
VIP
7PL
MIP
IPS1
IP2
IP0
V7
V3B
V3CD
LO2
TPOJ1
TPOJ2
MT
MST
FST
PH
PIT
V4
V8
FFC
TE2p
VVC
LO1
LO3
V4t
PGp
TPOJ3
STV
p24pr
a24pr
p32pr
a32pr
6mp
6ma
SFL
8BM
FEF
55b
IFSp
ventral visual areas (VV)
lateral visual areas (LV)
intraparietal sulcus areas (IPS)
inferior parietal areas (IP)
motor - somatosensory areas (mot-som)
supplementary motor areas (SMA)
premotor areas (PM)
anterior insular areas (AI)
inferior frontal areas (IF)
