# Supplementary figures and images for: Neuronal populations across the cortex underlie discrete, categorical, and subjective representations of visual durations
Source: PLoS Biol. 2026 Mar 26;24(3):e3003704. doi: 10.1371/journal.pbio.3003704 (PMC13020800; doi:10.1371/journal.pbio.3003704)

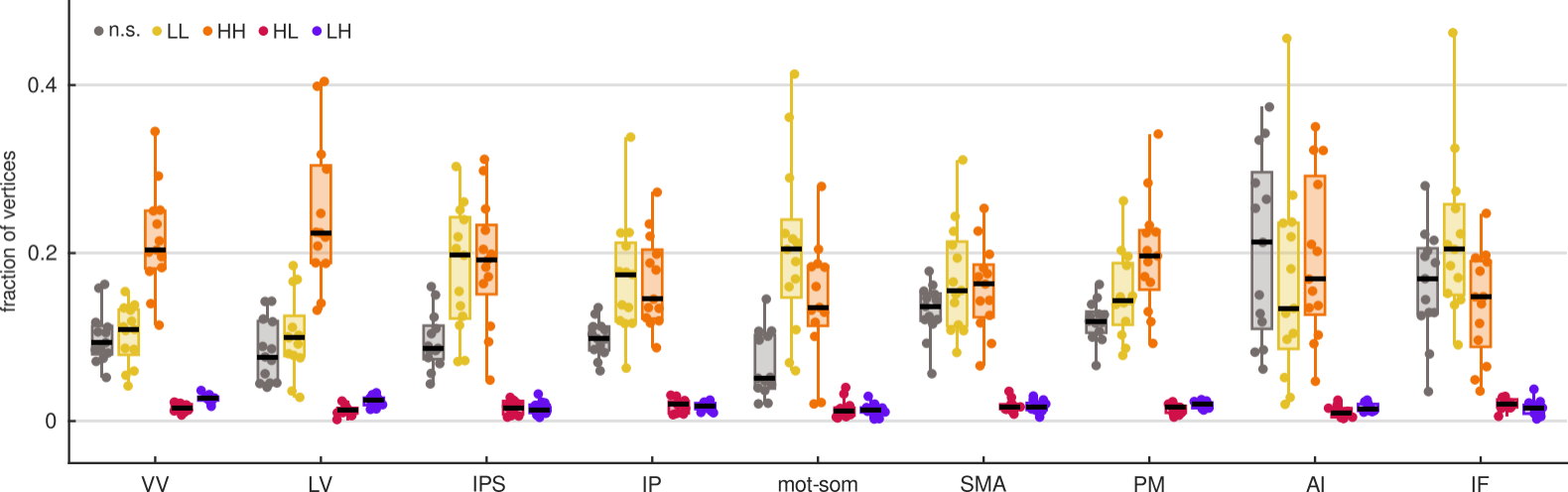

Supplement: S2 Fig — Box plots show the group-level distribution (n = 13) of the individual fraction of vertices assigned to each type of local spatial association (based on Moran’s I statistic) across the different functional streams. Spatial association types are color-coded: gray for non significant (n.s.), yellow for low-low (LL), orange for high-high (HH), pink for high-low (HL), and purple for low-high (LH). Each dot represents the mean fraction across ROIs and hemispheres for each participant. The horizontal black line indicates the median of the distribution, the box shows the interquartile range, and whiskers represent the minimum and maximum values. Streams are color-coded on the x-axis: green for ventral visual areas (VV), blue for lateral visual areas (LV), violet for intraparietal sulcus (IPS), purple for inferior parietal areas (IP), red for motor and somatosensory areas (mot-som), brown for supplementary motor areas (SMA), orange for premotor areas (PM), ochre for anterior insula (AI), yellow for inferior frontal areas (IF). See Methods - Analysis of duration preference categories along the cortical hierarchy - Local Moran’s I. Source data are available at the following link: osf.io/2tequ. (PDF) [file pbio.3003704.s002.pdf]

**a**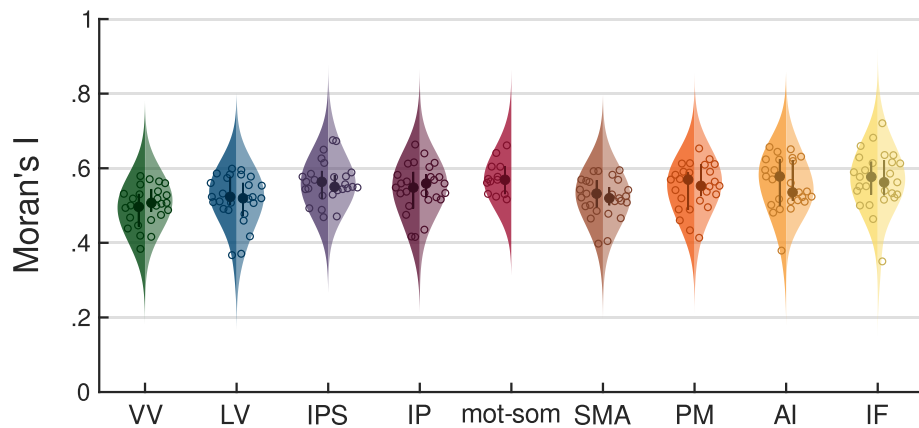**b**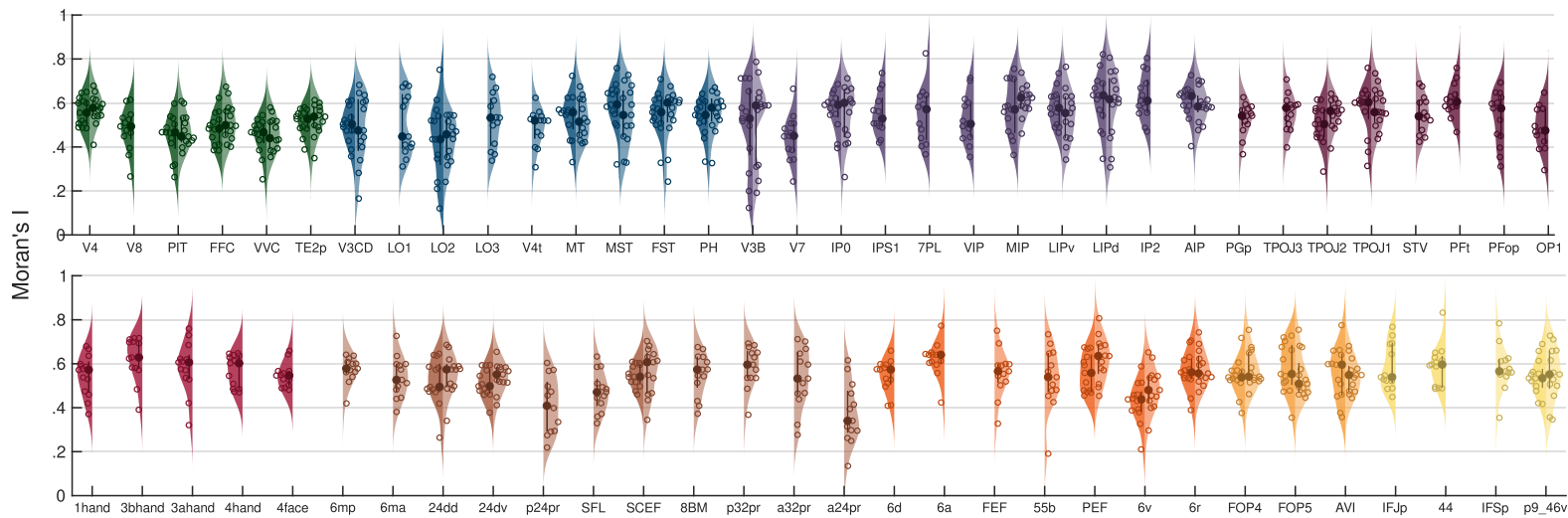

Supplement: S3 Fig — Each violin plot represents the group-level distribution (n = 13) of Moran’s I statistic values across streams (A) and ROIs (B). Both streams and ROIs are ordered from occipital to frontal and from dorsal to ventral. Streams are color-coded as follows: green for VV, blue for LV, violet for IPS, purple for IP, red for mot-som, brown for SMA, orange for PM, ochre for AI, yellow for IF. ROIs are color-coded according to their respective streams. The left side of each violin represents the left hemisphere (darker shades), while the right side represents the right hemisphere (lighter shades). Dots indicate the median of each distribution, while circles correspond to individual data points. Thick lines represent interquartile ranges. The kernel density estimates were computed using a 7% bandwidth. See Methods - Analysis of the topographic organization of duration preferences along the cortical hierarchy - Global Moran’s I. Source data are available at the following link: osf.io/2tequ. (PDF) [file pbio.3003704.s003.pdf]

**a**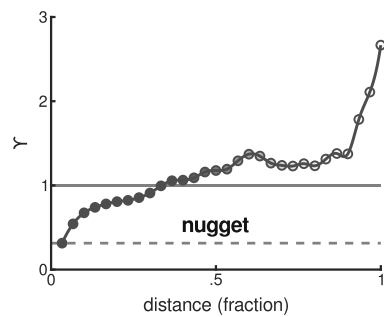**b**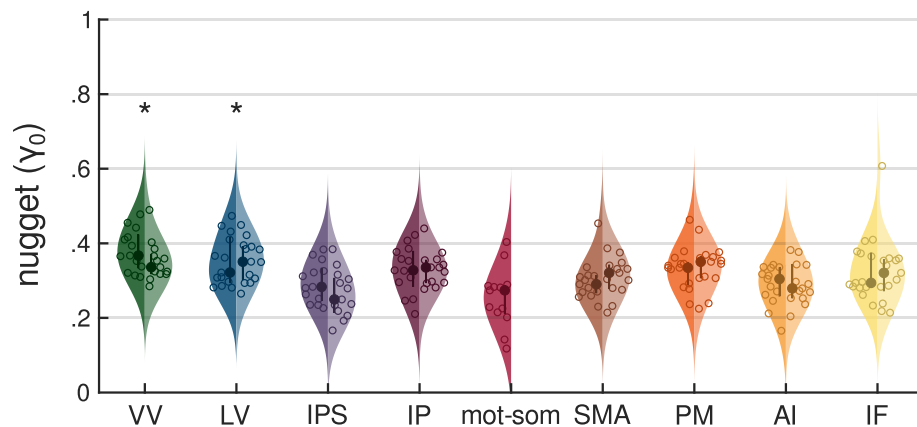**c**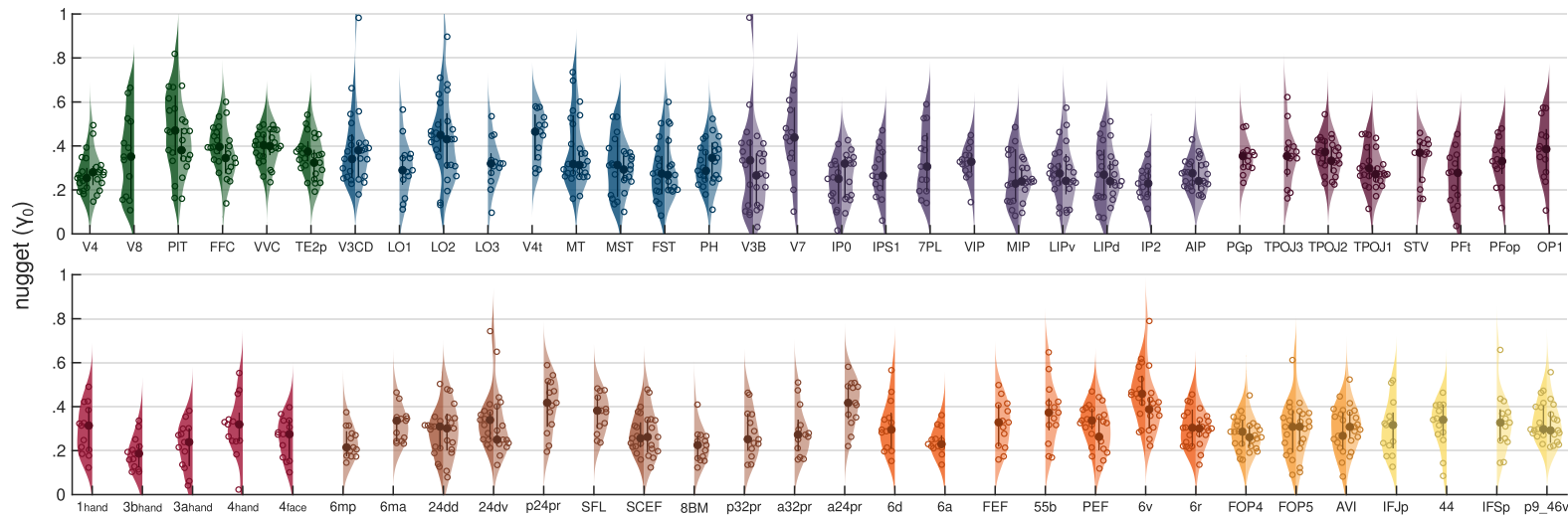

Supplement: S4 Fig — (A) An example variogram highlighting the nugget is shown. Each dot represents the variance (γ) in duration preferences between pairs of vertices at increasing distance (expressed as a fraction of the total extent of the ROI). Dots shading reflects the number of vertex pairs at each distance, with darker shades indicating a higher count. The solid line (γ = 1) indicates the total variance of the ROI, computed without accounting for spatial structure. The nugget (i.e., the variance at the shortest distance) is highlighted by the dashed line. Panels (B) and (C) show the group-level distributions (n = 13) of variogram nuggets across streams and ROIs, respectively. Nugget values are expressed as a fraction of the total variance of the ROI. Graphical details are the same as in S3 Fig. Asterisks in panel B indicate streams that are statistically different from the others (see S26 Table). See Methods - Analysis of the topographic organization of duration preferences along the cortical hierarchy - Variogram. Source data are available at the following link: osf.io/2tequ. (PDF) [file pbio.3003704.s004.pdf]

**a**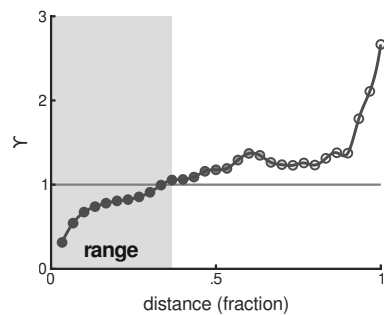**b**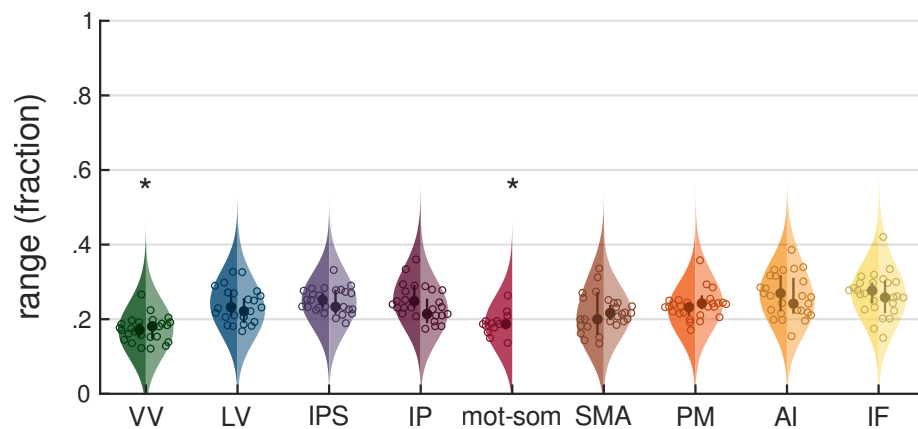**c**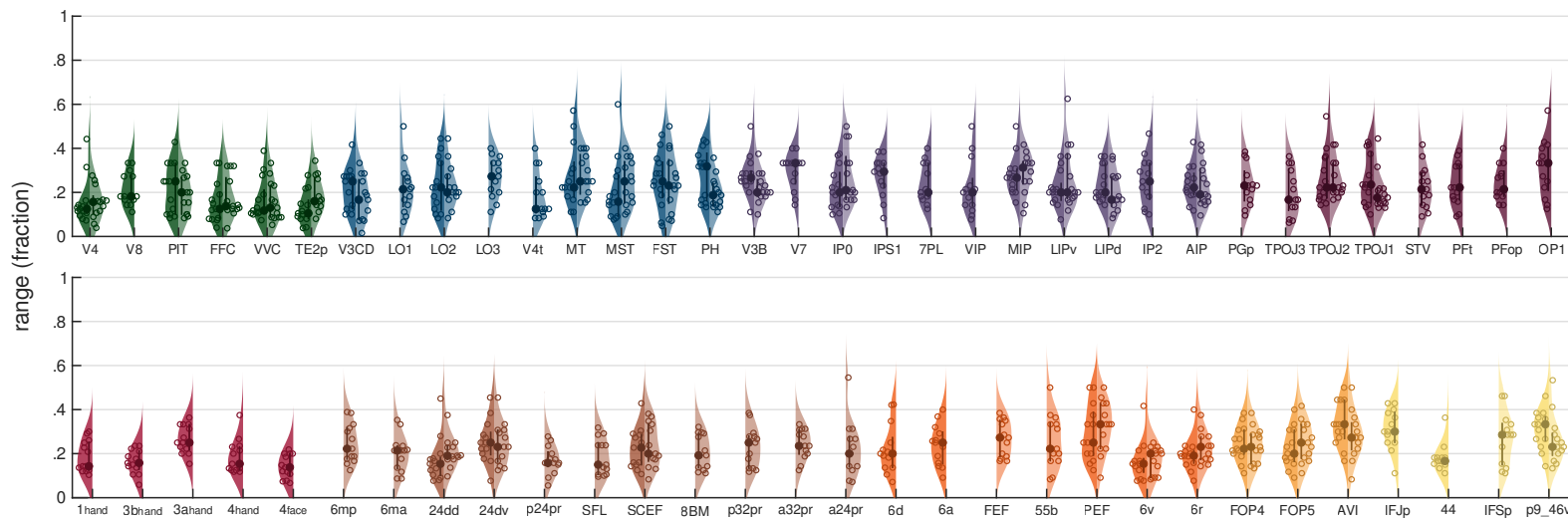

Supplement: S5 Fig — (A) The same example variogram from S4 Fig is shown, with the range (i.e., the distance at which the total variance of the ROI is reached) highlighted by a gray box. Panels (B) and (C) show the group-level distributions (n = 13) of variogram ranges across streams and ROIs, respectively. Range values are expressed as a fraction of the maximum distance between vertices of the ROI. Graphical details are the same as in S3 Fig. Asterisks in panel B indicate streams that are statistically different from the others (see S27 Table). See Methods - Analysis of the topographic organization of duration preferences along the cortical hierarchy - Variogram. Source data are available at the following link: osf.io/2tequ. (PDF) [file pbio.3003704.s005.pdf]

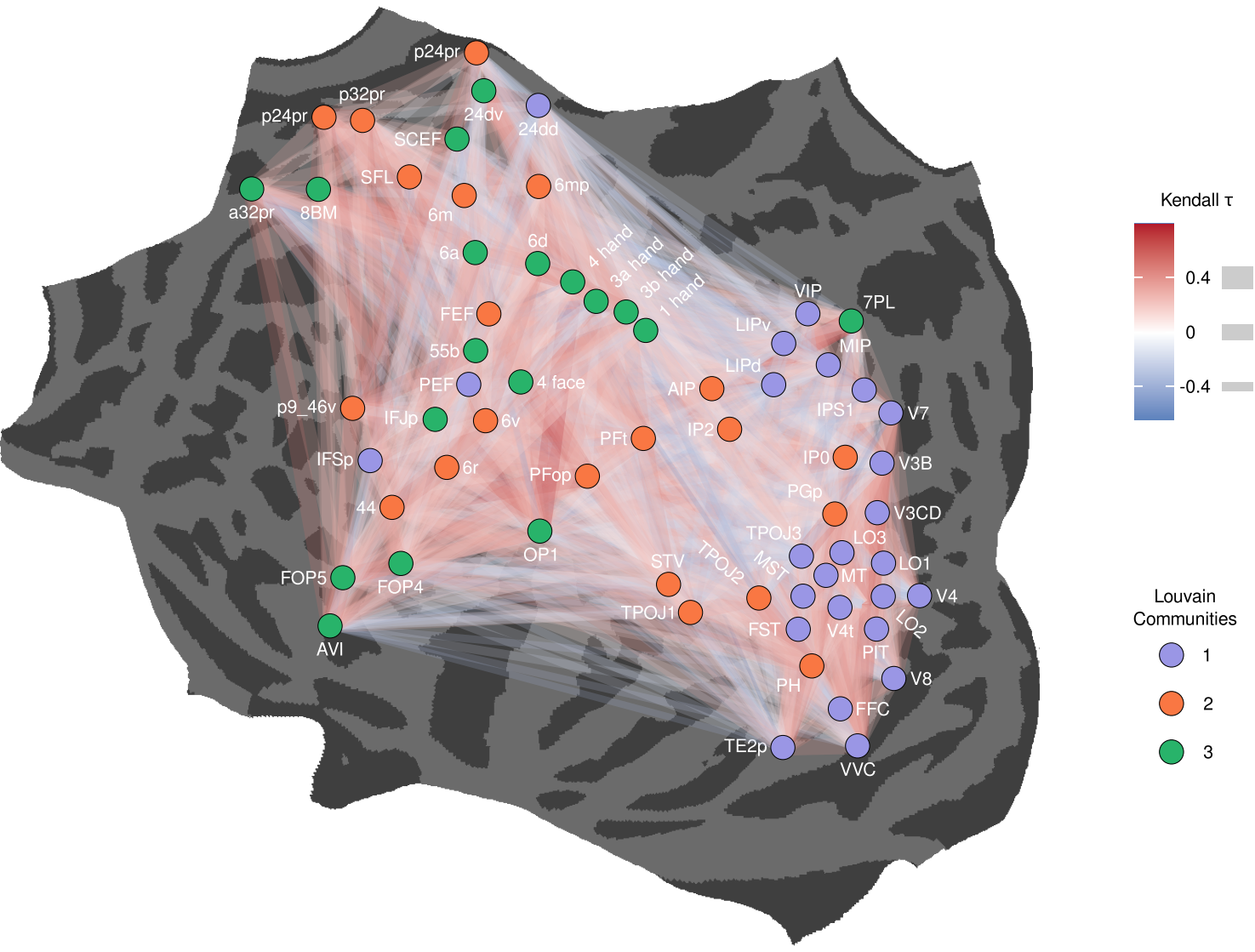

Supplement: S6 Fig — The group-level Kendall’s τ correlations, computed using the median preferred duration of each ROI and participant, are displayed on the cortical surface (fsaverage). To ease visualization, results are merged onto one hemisphere only. Each ROI is represented as a network node, and the color and width of edges connecting nodes indicate the corresponding correlation values. Node color represents the cluster to which each node belongs. Clusters were computed using the Louvain community detection algorithm. The results of this analysis, along with additional network quantification, are available in the OSF repository of this article as Gephi and Gephi Lite GEXF and JSON files. See Methods - Analysis of long-range relationships between duration preferences. (PDF) [file pbio.3003704.s006.pdf]
