# Supplementary material for: Neuronal populations across the cortex underlie discrete, categorical, and subjective representations of visual durations
Source: PLoS Biol. 2026 Mar 26;24(3):e3003704. doi: 10.1371/journal.pbio.3003704 (PMC13020800; doi:10.1371/journal.pbio.3003704)
Supplement: Attachment — Submitted filename: response_to_reviewers.pdf [file pbio.3003704.s038.pdf]

We sincerely thank the reviewers for their careful assessment of our manuscript and for their positive remark. We greatly appreciate their constructive suggestions to improve the clarity and quality of the manuscript, and to discuss or explore aspects that were previously left aside. In this point-by-point reply, we have tried our best to address the reviewers' comments and questions and to revise the manuscript accordingly. Our responses in this document, as well as changes in the main text and supplementary materials, are in teal font. Please also note that all line and figure numbers mentioned in this response refer to the revised version of the manuscript.

Reviewer #1

Centanino, Fortunato and Bueti present a manuscript of a re-analysis of data from a previously published report (Centanino, et al. 2024) in which human subjects (n=13) performed a time discrimination task using visual stimuli at distinct spatial locations and sub-second stimuli while being scanned at 7T. While the previous study focused on the dissociation between spatial position and temporal processing, this one instead is focused on tuning properties for durations, as an extension of previous work from this lab on gradient activation. The authors take a population receptive field modeling approach, in which the activity of vertices is analyzed by comparing to a Gaussian distribution at each of the presented durations to determine the maximally-responsive duration for each vertex, thereby determining which regions are most active to a particular interval or interval range. Across a series of analyses, the authors demonstrate that duration tuning appears to progress from early visual areas to parietal, somato-motor and frontal regions in a long-to-short pattern, with posterior regions favoring longer intervals and anterior regions favoring shorter ones, with midline regions such as the SMA and insula favoring the middle of the duration range tested. The authors examine this in various ways using various methods to examine the robustness of their findings, with a final link to behavior by demonstrating that biases in responses can be linked to activity in premotor, SMA, insula, and parietal regions. The authors conclude that early visual areas are monotonically linked to duration encoding, whereas midline areas such as the SMA are linked to the categorical boundary or middle interval and prefrontal regions to response preparation. Overall, the findings provide a rich and diverse set of data with some very interesting findings. The authors have taken great care in the preparation, curation, and analysis of this dataset, and it offers a nice complement to their previous paper in 2024. There are, however, some questions that I have regarding the findings as well as the presentation that I think would strengthen the manuscript, or at least provide some clarity to readers going forward.

1) The analyses are fairly complicated and so some effort at "unpacking" is helpful here. I think the authors have done a good job but the presentation can get muddled at times. In particular, I found Figure 2 confusing. The authors note that these are maps of duration preferences within each given region, from which I interpret the dominant blue shadings in early visual areas and red shadings in frontal areas to reflect the mean duration preferences for these regions as displayed in Figure 3, yet what do the individual pixels represent? That is, what are the x and y axes here? Is each region flattened/reduced to the same size? Why are some pixels white for some subjects and not others?

We thank the reviewer for this comment and agree that the information originally provided was insufficient to fully understand Figure 2. With pRF modeling, we estimated a duration preference for each vertex of the entire cortical surface of each participant. In Figure 2, we aimed to show how these duration preferences are spatially arranged across the cortical surface of a selection of our regions of interest (ROI). Accordingly, each box of Figure 2 represents the cortical space of a given ROI (columns) for a given participant (rows). To facilitate visualization, we adopted two strategies. First, we flattened the cortical surface to allow representation in a two-dimensional plane. Second, we resampled the cortical space of each ROI onto an isotropic two-dimensional grid of 2 mm resolution. To represent the duration maps, we computed for each grid pixel the average duration preference of ROI vertices weighted by a Gaussian filter with a full width at half maximum of 4 mm and centered on that pixel. As a result, the x- and y-axes of each box in Figure 2 correspond to the x and y coordinates of this grid, and each pixel represents a smoothed preferred duration, color-coded according to the colorbar shown at the bottom of the figure. This process yielded grid sizes that varied across participants and ROIs, reflecting differences in the original size of each region. Some pixels are white because each individual region has an irregular and unique shape, and fitting it within the grid resulted in unpopulated pixels. We adopted this procedure solely to make duration maps visually comparable across regions and participants. We have now added this information to the caption of Figure 2, which reads as follows:

Duration preference maps are shown for all participants (in separate rows) across a selection of ROIs (ordered from occipital to frontal in the different columns), in either the left (LO2, 7PL, MIP, TPOJ2, 3a_hand, AVI) or the right (24dv, a32pr) hemisphere. Each map represents the cortical space of a given ROI, flattened and resampled onto an isotropic two-dimensional grid (2 mm resolution). The color of each grid cell represents the average duration preference (from red, 0.2 s, to blue, 0.8 s) of the ROI vertices weighted by a Gaussian kernel (full width at half maximum = 4 mm) centered on that cell. Maps belonging to the same functional stream are grouped with a color-coded outline (blue for LV, violet for IPS, purple for IP, red for mot-som, brown for SMA, ochre for AI). Black lines indicate isolines that join locations with

equal preference values. White pixels correspond to locations outside of the ROI.

2) The local Moran's statistics is interesting, but I'm not sure why there would be a lack of HH and LH associations. If the statistic looks for vertices that are completely surrounded by one type of duration tuning them perhaps a lack of LH isn't surprising (a low center surrounded by high duration preferences), but why would there not be any HH associations? I would expect these to be found in visual areas as found using other measures.

We thank the reviewer for this question, which allows us to clarify the rationale for using the local Moran's I statistic. First, it is important to note that we employed this statistic to test the *number* of spatial association types across regions, and not their *nature* (lines 234-238, "We hypothesized that brain regions selective to one specific duration category (i.e., VV, LV, mot-som, rostral SMA, AI, IF) would also show one dominant type of spatial association between neighboring vertices. On the other hand, regions where all duration categories are equally represented (i.e., IPS, IP, caudal SMA) should show multiple spatial association types."). We deliberately avoided interpreting the nature of the observed spatial associations because it strongly depends on (i) the chosen neighboring structure and (ii) the fact that the variable used for the statistic (i.e. duration preference in our case) is expressed as deviation from the sample mean. Indeed, the formula of the local Moran's I for a variable x at location i with neighbors j is:

$$I_i = c * z_i \sum_j w_{ij} z_j \tag{1}$$

where $z_i = x_i - \overline{x}$, $z_j = x_j - \overline{x}$, $c = \frac{1}{\sum_i z_i^2}$, and w_{ij} are the spatial weights. In our analysis, we computed the local Moran's I statistic across the entire cortical surface of each hemisphere (i.e., not ROI by ROI), using a broad neighboring structure (59-135 vertices) and centering duration preferences on the mean of that hemisphere (see *Methods - Analysis of duration preference categories along the cortical hierarchy - Local Moran's I*). This approach makes the statistic sensitive to macroscopic, coarse-grained patterns of spatial associations across the cortex, complementing the categorization analysis of duration preferences (see *Results - Categorization of duration preferences along the cortical hierarchy*), but limits the ability to assess their specific nature, which would instead require a more fine-grained and ROI-based analysis (i.e., restricting the computation to duration preferences within each ROI, tailoring the neighboring structure to the size of each ROI, and centering duration preferences on the ROI mean). However, such an ROI-based approach would make the results not directly comparable across ROIs. For these reasons, we opted for a macroscopic analysis and intentionally omitted interpretation of the specific spatial association types. Accordingly, the absence of some association types should not be interpreted in absolute terms, but rather as a byproduct of this methodological choice. Nevertheless, we were also puzzled by the systematic absence of HH and LH associations, and during our analysis we could not identify an obvious explanation. Prompted by the reviewer's question, we went back to our code and, unfortunately, found the explanation there. Due to a bug, the computation of the number of HH vertices was collapsing into the computation of the number of HL vertices. We report below the function we used to compute local Moran's I spatial associations to better clarify what happened.

```
function clusters = moran_clustering(p_vals,mu,mu_neighbors,alpha)

if nargin < 4 || isempty(alpha)
    alpha = 0.05;
end

%MORAN_CLUSTERING groups the vertices with a significant local Moran's I
%into low-low, high-high, high-low, and low-high clusters.
%INPUTS:            p_vals: Nx1 vector of pvalues of local Moran's I, where N is the number of vertices
%                   mu: Nx1 vector of mu values (centered), where N is the number of vertices
%                   mu_neighbors: NxM matrix of mu values (centered) of neighbors, where N is the number of vertices and M is the number of neighbors
%                   alpha: significance threshold [default: 0.05]
%OUTPUT:            clusters: Nx1 vector of clusters ID for each vertex
%                             0 -> not significant (NS)
%                             1 -> low-low (LL)
%                             2 -> high-high (HH)
%                             3 -> high-low (HL)
```

```
18 %                               4 -> low-high (LH)
19
20 to_skip = isnan(mu) | all(isnan(mu_neighbors),2);
21 clusters = nan(size(to_skip));
22
23 high_mu = mu(~to_skip) > 0;
24 high_neighbors = mean(mu_neighbors(~to_skip,:),2,'omitnan') > 0;
25 valid_clusters = zeros(size(high_mu));
26 valid_clusters(~high_mu & ~high_neighbors) = 1; % LL
27 valid_clusters(high_mu & high_neighbors) = 2; % HH
28 valid_clusters(high_mu & ~high_neighbors) = 3; % HL
29 valid_clusters(~high_mu & high_neighbors) = 4; % LH
30 pvals_mask = p_vals(~to_skip) < alpha;
31 valid_clusters = valid_clusters.*pvals_mask; % NS
32
33 clusters(~to_skip) = valid_clusters;
```

In the function above, line 24 defines whether neighbors are, on average, low or high. Since at this stage all duration preferences are already centered on the sample mean, this corresponds to testing whether neighbors are, on average, positive or negative. Previously, this line was erroneously run without the `'omitnan'` flag. Because we do have NaNs for vertices that did not show a proper pRF fit (see *Methods - Population Receptive Field (pRF) modeling*), including them in the average caused the mean of some neighbors to be a NaN too, which never counted as positive in line 24. This drastically reduced the number of associations with high neighbors (only vertices without NaNs among neighbors were properly labeled as high) and inflated the number of associations with low neighbors. After recomputing the associations correctly (i.e., with the `'omitnan'` flag), the two prevailing spatial associations are LL and HH, as expected from the data. Essentially, all HH associations had previously been misclassified as HL, while the other association types remained largely unchanged. Indeed, the pattern of differences between LL and HH associations (Figures 4b and 4d, Supplementary Figure S2) and the corresponding statistics (lines 245-261 and Supplementary Tables S16 - S23) remain consistent with the original results. While it remains true that the nature of spatial associations cannot be interpreted in absolute terms, finding LL and HH as the dominant types, and observing that they swap across the cortical hierarchy, is now fully consistent with the other results and with visual inspection of the data. We sincerely apologize for this oversight, which was largely due to differences in how MATLAB treats NaNs across versions, and we are grateful to the reviewer for prompting us to identify and correct it. In addition to reporting the updated results in the manuscript, we have now clarified in the Methods section (lines 823–827) that our implementation of the Moran's I statistic does not allow for a faithful characterization of the nature of spatial associations. The text now reads as follows:

This approach allowed us to capture macroscopic patterns of spatial associations of duration preferences and to compare them across the cortex, at the expense of resolving fine-grained spatial patterns within each ROI. Therefore, the observed proportions of spatial association types should not be interpreted in absolute terms, but rather as a byproduct of this methodological choice.

3) The link to the behavioral data is the most interesting to me, as it provides a nice connection to how these areas are associated with perceptual experience. However some questions remain: first, did the authors explore any other behavioral measures here? The coefficient of variation (CV) could be just as informative as the PSE here, by demonstrating how the variance or "noisiness" of the representation is affected by the tuning properties of individual regions/subjects. Second, if the prefrontal regions are indeed reflecting motor preparation then one would expect a link here with reaction time (RT). I recognize that the task was not speeded, yet the authors could see if 1) RTs nonetheless differed as a function of duration, and 2) activity in the frontal regions correlates with the speed of response, thus supporting their argument.

We agree with the reviewer that the link between tuning and behavior would benefit from a more comprehensive investigation, and that correlating measures of tuning and perceptual "noisiness" is an interesting direction. Indeed, in earlier iterations of our analyses, we explored this possibility by using the Just Noticeable Difference (JND) as a measure of perceptual sensitivity. We decided to use the JND instead of the CV, as suggested by the reviewer, because CV computed as $\frac{JND}{reference}$ would merely rescale the JND and not add information; on the other hand, CV computed as $\frac{JND}{PSE}$ would introduce a potential confound, since the PSE was found to correlate with the pRF mean. The neural counterpart of sensitivity is the sigma parameter of the pRF tuning function, which directly describes how narrow or broad brain responses are. However, our experiment is not well suited for a reliable estimation this parameter, and for this reason we chose not to correlate it with the JND. First and most importantly, the sigma of a Gaussian pRF model is known to be less reliably estimated than the mean and to be generally more sensitive to fMRI noise[1]. This limitation is further amplified in our

case because the tuning model we used is two-dimensional (one dimension for stimulus duration and the other for stimulus spatial position). This is evident in Figure 1b, which shows the model fit for one example vertex. For the same duration, the signal fluctuates across spatial positions, affecting the estimation of the sigma more than the estimation of the mean. For this reason, we opted not to use these sigma estimates directly. Instead, we correlated the JND with the interquartile range of the duration preference distributions of each stream and ROI, reasoning that the spread of these distributions could still provide an indirect index of the overall sensitivity/noisiness of duration tuning. Figure R1 shows the results across streams (a) and ROIs (b). At the stream level, we did not observe any significant effects, whereas at the ROI level, we found positive correlations in MIP, 6ma, and 6a.

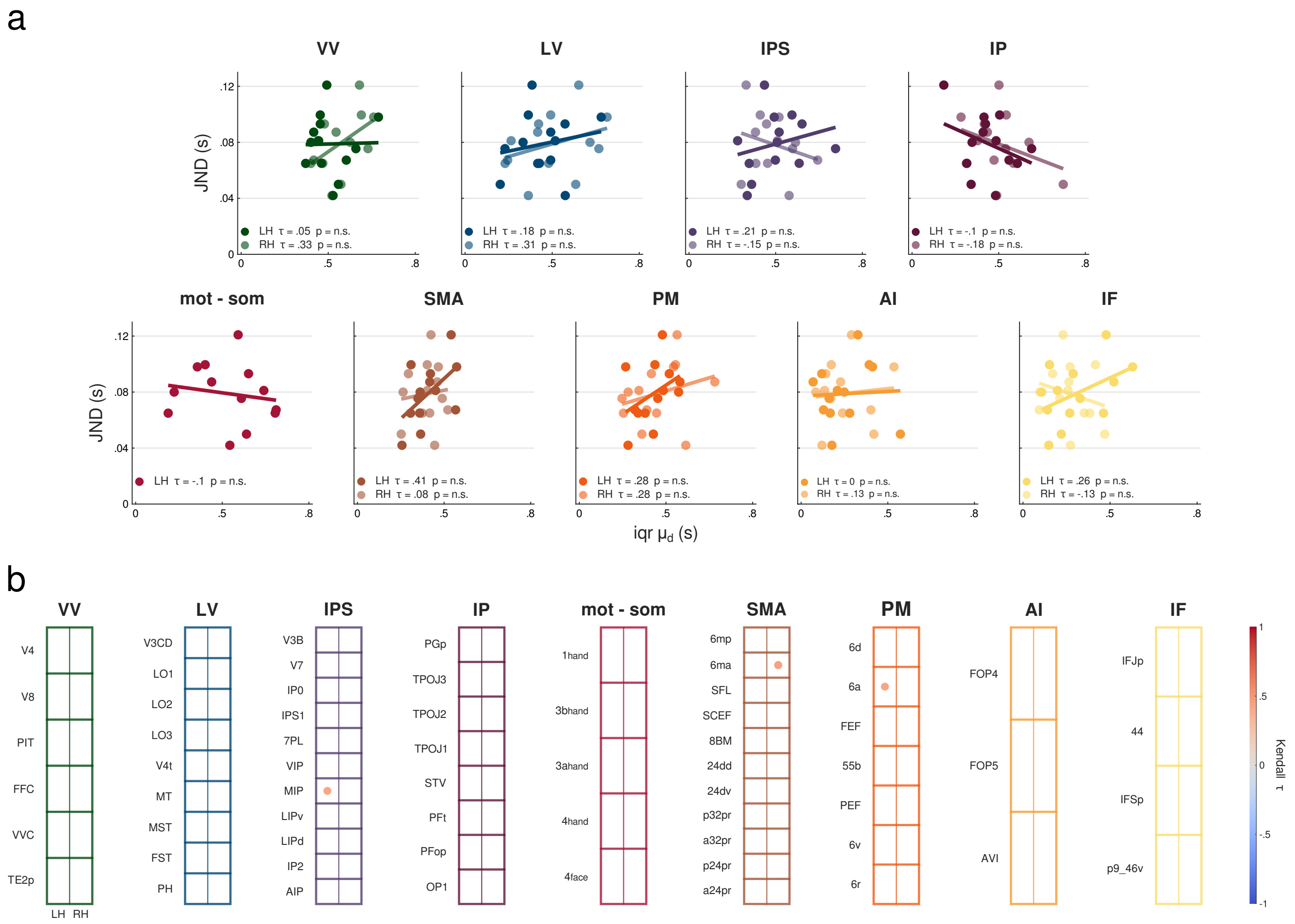

Figure R1: **Correlation between JND and duration tuning.** **(a)** Each scatter plot corresponds to a different stream, where individual JND values (y-axis) are plotted against individual interquartile ranges of preferred durations (x-axis) of that stream along with regression lines. Darker dots refer to the left hemisphere, while lighter dots refer to the right hemisphere. Kendall τ correlation values and their associated p-values are reported in the bottom left corner of each plot. **(b)** Each grid represents a different stream, with ROIs arranged in rows. The left (LH) and right (RH) hemispheres are displayed in separate columns. Each dot represents the Kendall correlation between individual JND values and individual interquartile ranges of preferred durations for a given ROI. Dot color indicates the Kendall τ value. Only significant (p-value < 0.05) correlations are shown.

We ultimately decided not to include these results in the manuscript for several reasons. First, our experimental design was not optimized for a robust estimation of participants' sensitivity. The temporal task was intentionally kept very easy to facilitate performance inside the MRI scanner (absolute differences from reference and comparisons were 100, 200, and 300 ms). In addition, we used six comparison durations, and the reference duration itself was not included among them, resulting in a JND estimate based on relatively few data points. Second, the relationship between tuning parameters and behavioral performance remains largely unexplored in the fMRI literature. While the link between PSE and duration preferences was consistent with electrophysiological findings in monkeys[2], for the JND we lacked a strong theoretical or

empirical reference. Third, our correlation analysis was based on a relatively small sample (13 participants) and under these circumstances a correlation observed consistently at both the stream and ROI levels, as we did for the PSE, would have been more robust; however, this was not the case. Overall, we adopted a conservative approach as we believe that the relationship between tuning and behavior should be addressed with experiments specifically designed to probe it both neurally and behaviorally. Indeed, we are currently running two fMRI studies explicitly aimed at investigating this link (see e.g., [3]).

Concerning the second point raised by the reviewer, it is important to specify that the areas we linked to motor preparation are motor and somatosensory regions, primarily associated with hand representation (1_hand, 3a_hand, 3b_hand, 4_hand, 4_face). Nonetheless, we acknowledge that this interpretation is based on the known functional roles of these areas and on the observation that their activation was exclusively contralateral to the hand used by participants to perform the task. The reviewer's suggestion to examine the relationship between brain activity and reaction times (RTs) therefore provides a potentially powerful way to further support our claim. Figure R2 shows the group-level distribution of RTs across the different presented durations.

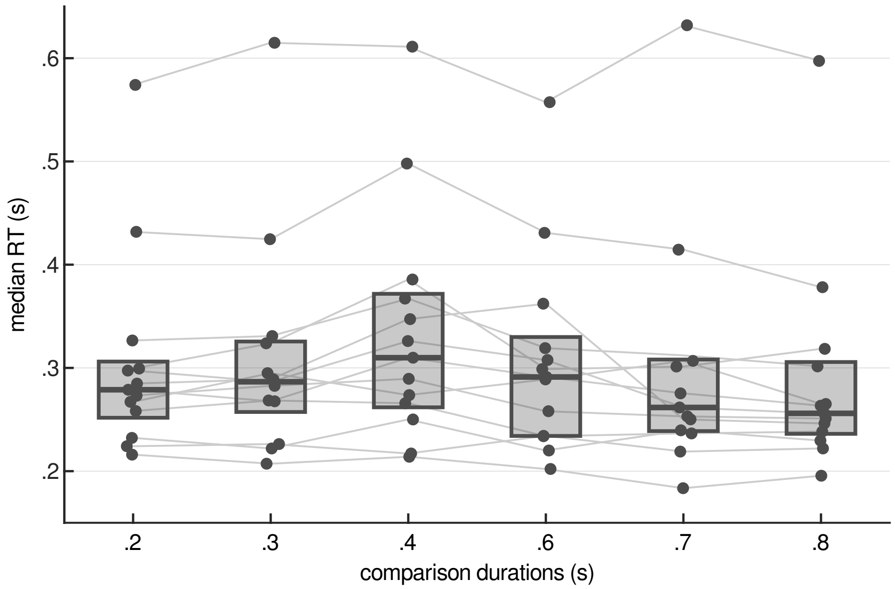

Figure R2: **Reaction times (RTs) across comparison durations.** Box plots show the group-level distribution of RTs across the different comparison durations. Dots indicate each participant's median RT, and thin gray lines connect dots from the same participant. Within each box plot, the thick horizontal line represents the median and the box indicates the interquartile range.

To assess RT changes as a function of duration, we tested a linear mixed effects (LME) model with duration as factor and participant as random intercept (model formula: $log(RT) \sim duration + (1|subjectID)$, marginal R^2: 0.01, conditional R^2: 0.32). A Type III ANOVA on model estimates revealed a main effect of duration ($F(5,5333) = 15.92$ $p < 0.0001$). Specifically, RTs tended to be slower for comparison durations closer to the reference ($t(5333) > 3.24$, $p < 0.05$ comparing 0.4 s with 0.2 s and 0.3 s; $t(5333) = 4.78$, $p < 0.0001$ comparing 0.6 s with 0.8 s). In addition, RTs for durations shorter than the reference were generally slower than those for longer ones ($t(5333) > 3.26$, $p < 0.05$ comparing 0.4 s with 0.6 s, 0.7 s and 0.8 s; $t(5333) = 4.83$, $p < 0.0001$ comparing 0.3 s with 0.8 s). Together, these results indicate that RTs vary systematically with the difficulty of the temporal task. The full set of statistics is reported in tables R1 and R2.

	Sum.Sq	Mean.Sq	NumDF	DenDF	F.value	Pr(>F)
duration	16.11	3.22	5	5333	15.92	1.34E-15

Table R1: **Reaction times (RTs) across comparison durations.** Type III ANOVA on LME model estimates with Satterthwaite's method for degrees of freedom. RTs were log-transformed to improve normality and ensure more robust performance of the LME model.

contrast	estimate	SE	df	t.ratio	p.value
0.2s - 0.3s	-0.05	0.02	5333	-2.56	1.59E-01
0.2s - 0.4s	-0.12	0.02	5333	-5.81	9.86E-08
0.2s - 0.6s	-0.05	0.02	5333	-2.52	1.78E-01
0.2s - 0.7s	0.00	0.02	5333	0.22	1.00E+00
0.2s - 0.8s	0.05	0.02	5333	2.30	3.23E-01
0.3s - 0.4s	-0.07	0.02	5333	-3.24	1.78E-02
0.3s - 0.6s	0.00	0.02	5333	0.03	1.00E+00
0.3s - 0.7s	0.06	0.02	5333	2.74	9.11E-02
0.3s - 0.8s	0.10	0.02	5333	4.82	2.16E-05
0.4s - 0.6s	0.07	0.02	5333	3.26	1.66E-02
0.4s - 0.7s	0.13	0.02	5333	5.96	3.92E-08
0.4s - 0.8s	0.17	0.02	5333	8.05	1.55E-14
0.6s - 0.7s	0.06	0.02	5333	2.70	1.03E-01
0.6s - 0.8s	0.10	0.02	5333	4.78	2.72E-05
0.7s - 0.8s	0.04	0.02	5333	2.06	5.88E-01

Table R2: **Reaction times (RTs) across comparison durations.** Two-sided t contrasts between comparison durations. Contrast estimates are expressed as difference of log scaled values. All p values are Bonferroni corrected for multiple comparisons.

We then checked whether brain activity in motor and somatosensory areas (i.e., 1_hand, 3a_hand, 3b_hand, 4_hand, 4_face) relates to RTs. To this end, we first performed a single-trial GLM analysis using the GLMsingle toolbox[4]. For each trial, we modeled activity using a single regressor defined as a boxcar covering the shortest possible stimulus–cue interval (SCI). This approach allowed us to capture both stimulus-driven and response-related activity. To assess whether single-trial brain activity varied as a function of RTs, we tested a LME model with RT as factor and random slope, and participant and area as random intercepts ($\beta \sim log(RT) + (log(RT)|ID) + (1|subjectID)$, marginal R^2: 0.00055, conditional R^2: 0.102). Type III ANOVA on model estimates revealed a main effect of RT ($F(1,8.77) = 6.09$ $p = 0.0363$). This result indicates that lower brain activity is linked to longer RTs (estimated slope = -0.0148, s.e. = 0.006). However, it is important to note that the overall performance of the model is very low, and the explanatory power of RTs is negligible. This is likely due to the inherently noisy nature of single-trial beta estimates in fMRI, particularly in fast event-related designs with brief events such as the present one. In addition, RTs in our experiment are not purely related to the temporal task, as we introduced a jittered delay between stimulus offset and the response cue (i.e., the SCI). Therefore, although this analysis provides some support for our interpretation, the results should be interpreted with caution. The full statistics is reported in table R3.

	Sum.Sq	Mean.Sq	NumDF	DenDF	F.value	Pr(>F)
log(RT)	0.61	0.61	1	9	6.09	3.63E-02

Table R3: **Brain activity (single-trial β) across reaction times (RTs).** Type III ANOVA on LME model estimates with Satterthwaite's method for degrees of freedom. RTs were log-transformed to improve normality and ensure more robust performance of the LME model.

We now refer to this analysis in the manuscript (Results, lines 217-220) as follows:

In motor and somatosensory areas, the selectivity for short durations is likely a by-product of motor preparation responses, as supported by the observed negative relationship between brain activity preceding the response and reaction times (see S13 - S15 Table and Discussion).

4) Connected to the above, the authors don't go into any detail regarding the negative correlations that are observed here. That is, there appears to be a negative correlation between VVC and V3CD and PSE, such that higher activity in these regions corresponds to shorter perceived durations, which seems opposite to the argument the authors make that activity in these regions reflects monotonic increases with longer durations.

As discussed in point 3, we adopted a conservative approach in the correlation analysis for several reasons. First, this is, to our knowledge, the first attempt to link pRF tuning parameters and behavioral measurements in humans. Second, our task was designed to ensure robust performance in the scanner rather than to finely probe duration perception. Third, correlations were computed on a relatively small sample (13 participants). For these reasons, we acknowledge that our results warrant a cautious interpretation. Accordingly, we used Kendall's correlation, which is more robust to outliers,

and we restricted our discussion to correlations that (i) were observed consistently at both stream and ROI levels and (ii) were supported by electrophysiological findings. In addition, correlations between tuning and behavior in occipital areas require even more careful interpretation. In fact, duration tuning in these regions has been shown to follow a compressive monotonic, rather than Gaussian, function[5]–[8]. As discussed in the manuscript, by employing a Gaussian model we nonetheless capture this type of tuning, and indeed we observed a predominance of long duration preferences in occipital visual areas, consistent with stronger responses to longer durations. However, a proper investigation of the relationship between duration perception and monotonic tuning would require explicitly modeling brain responses with a monotonic function, which was beyond the scope of the present study. Nevertheless, we do have a hypothesis regarding the observed negative correlations, whereby longer PSEs (i.e., shorter perceived durations) are associated with shorter preferred durations of the Gaussian model, and vice versa. In occipital visual areas, variations in duration preferences estimated via a Gaussian model may in fact reflect different degrees of compression of the underlying monotonic function. This is illustrated in Figure R3, where monotonic functions with varying degrees of compression (colored solid lines) are shown alongside their best-fitting Gaussian approximations (colored dashed lines). As compression increases (i.e., as the compressive exponent approaches zero), the mean of the corresponding Gaussian shifts toward shorter durations.

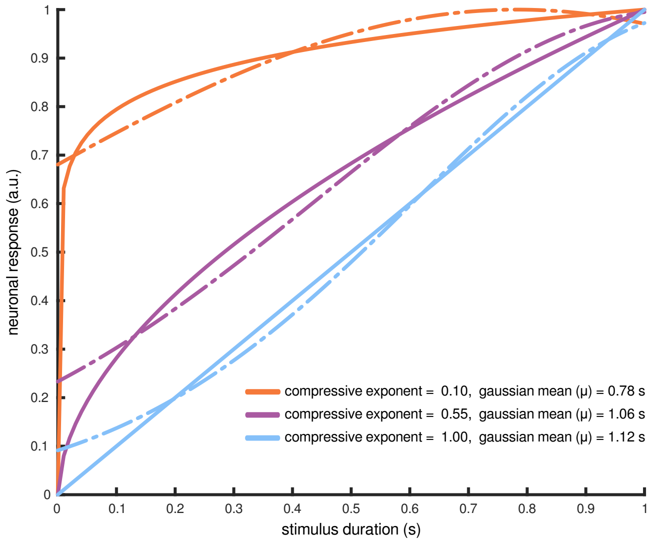

Figure R3: **Relationship between compressive monotonic and Gaussian functions.** Solid lines indicate monotonic functions with varying compressive exponents, and dashed lines represent their best-fitting Gaussian approximations. The compressive exponents and Gaussian means are reported in the legend.

Under this framework, the negative correlations we observed may indicate that participants who tend to underestimate durations (longer PSEs) also exhibit stronger saturation (shorter preferred durations) in certain visual regions. Compressive monotonic tuning for duration in visual areas has been interpreted as reflecting the accrual of sensory information, which is subsequently read out into a discrete duration through Gaussian tuning in downstream regions. Under this view, the amount of accumulated sensory information directly inform the subsequent stages of duration processing, and the saturation point can be seen as the capacity of a neural system to integrate sensory input. A system with lower capacity (i.e., stronger saturation) would systematically accumulate less information, leading to shorter represented durations and, eventually, behavioral underestimation. We are currently conducting experiments specifically designed to test this hypothesis. We acknowledge that this interpretation relies on multiple assumptions and introduces considerable complexity, which is why we chose not to include it in the manuscript. However, we agree that completely omitting mention of these negative correlations could appear superficial. For this reason, we have revised lines 453–462 of the Discussion, which now read as follows:

Our findings further support this view, suggesting that in the visual domain, monotonic responses may reflect an "accumulation" process of sensory information that encodes the duration of visual events. The negative correlation between duration tuning and behavior observed in specific ventral and lateral and visual areas (right VVC and left V3CD) points to a direct involvement of this accumulation mechanism in duration perception, although its precise interpretation would require targeted experiments and analyses. Within this framework, such an encoding mechanism could also account for the dilation of perceived time induced by the increase in low-level stimulus properties, such as speed, size, or visibility, by

positing that greater sensory evidence accumulation leads to longer encoded durations.

Minor comments:

5) Figure 4 is a little confusing in one regard: why are the lines curved? For example, in panel a there is an "arc" from VV to LV on the blue curve, but why would this be? Shouldn't it be a straight line?

We thank the reviewer for this observation, as we indeed did not explain what the lines in Figure 4 represent. These lines were obtained using a spline interpolation across data points, which is why they appear curved rather than straight. They were used solely to aid visualization and highlight the overall trend, particularly in panel (c), which is highly cluttered due to the presence of 64 ROIs. However, we acknowledge that these curved lines may be confusing, even with a proper explanation in the caption. For this reason, we have replaced them with straight lines connecting the data points. The updated figure and its corresponding caption are now included in the manuscript.

6) Given that so many correlations were run, both in Figure 6 and 7, were there any corrections for multiple comparisons?

For Figure 6, we did not consider the significance of the correlations because the goal of the analysis was to capture the overall structure of changes in duration preferences across the hierarchy. To this end, we transformed the full correlation matrix into a distance matrix and performed a hierarchical clustering analysis, using correlations to characterize the relative relationships between areas rather than to test their statistical significance. For Figure 7, we did not apply correction for multiple comparisons. Tables R4 and R5 show Bonferroni and FDR corrections performed on the full set of tests (i.e., 17 for streams; 93 for ROIs) for correlations at the stream and ROI levels, respectively.

Hemi	stream	tau	p	p_bonferroni	p_fdr
lh	VV	-0.077	0.765	1.000	0.929
lh	LV	-0.103	0.675	1.000	0.929
lh	IPS	0.154	0.510	1.000	0.929
lh	IP	0.436	0.042	0.717	0.239
lh	mot-som	0.359	0.100	1.000	0.283
lh	SMA	0.051	0.858	1.000	0.972
lh	PM	0.333	0.129	1.000	0.313
lh	AI	0.436	0.042	0.717	0.239
lh	IF	0.385	0.076	1.000	0.283
rh	VV	0.000	1.000	1.000	1.000
rh	LV	0.000	1.000	1.000	1.000
rh	IPS	-0.077	0.765	1.000	0.929
rh	IP	-0.359	0.100	1.000	0.283
rh	SMA	0.256	0.252	1.000	0.535
rh	PM	0.077	0.765	1.000	0.929
rh	AI	0.590	0.004	0.073	0.073
rh	IF	0.128	0.590	1.000	0.929

Table R4: **Multiple comparison correction of Kendall τ values between duration preference and PSE across streams.**

Hemi	ROI	tau	p	p_bonferroni	p_fdr
lh	V4	0.077	0.765	1.000	0.936
lh	V8	-0.077	0.765	1.000	0.936
lh	PIT	0.154	0.510	1.000	0.765
lh	FFC	0.103	0.675	1.000	0.860
lh	VVC	-0.359	0.100	1.000	0.664
lh	TE2p	-0.128	0.590	1.000	0.807
lh	V3CD	-0.462	0.030	1.000	0.436
lh	LO2	0.026	0.952	1.000	0.973
lh	MT	-0.308	0.163	1.000	0.724
lh	MST	-0.154	0.510	1.000	0.765
lh	FST	0.051	0.858	1.000	0.961
lh	PH	-0.051	0.858	1.000	0.961

lh	V3B	-0.256	0.252	1.000	0.765
lh	V7	0.026	0.952	1.000	0.973
lh	IP0	0.256	0.252	1.000	0.765
lh	IPS1	-0.154	0.510	1.000	0.765
lh	7PL	0.436	0.042	1.000	0.436
lh	VIP	0.103	0.675	1.000	0.860
lh	MIP	-0.026	0.952	1.000	0.973
lh	LIPv	0.179	0.435	1.000	0.765
lh	LIPd	-0.179	0.435	1.000	0.765
lh	IP2	-0.231	0.306	1.000	0.765
lh	AIP	0.205	0.367	1.000	0.765
lh	TPOJ2	0.128	0.590	1.000	0.807
lh	TPOJ1	0.308	0.163	1.000	0.724
lh	PFt	0.231	0.306	1.000	0.765
lh	PFop	0.410	0.057	1.000	0.484
lh	OP1	0.513	0.015	1.000	0.436
lh	1_hand	0.385	0.076	1.000	0.546
lh	3b_hand	0.179	0.435	1.000	0.765
lh	3a_hand	0.179	0.435	1.000	0.765
lh	4_hand	0.436	0.042	1.000	0.436
lh	4_face	0.026	0.952	1.000	0.973
lh	SCEF	0.154	0.510	1.000	0.765
lh	24dd	0.026	0.952	1.000	0.973
lh	24dv	0.026	0.952	1.000	0.973
lh	6d	0.308	0.163	1.000	0.724
lh	6a	0.154	0.510	1.000	0.765
lh	PEF	0.205	0.367	1.000	0.765
lh	6v	0.154	0.510	1.000	0.765
lh	6r	0.000	1.000	1.000	1.000
lh	FOP4	0.333	0.129	1.000	0.724
lh	FOP5	0.436	0.042	1.000	0.436
lh	AVI	0.487	0.022	1.000	0.436
lh	IFJp	0.308	0.163	1.000	0.724
lh	44	0.128	0.590	1.000	0.807
lh	p9_46v	0.333	0.129	1.000	0.724
rh	V4	-0.231	0.306	1.000	0.765
rh	PIT	-0.051	0.858	1.000	0.961
rh	FFC	0.154	0.510	1.000	0.765
rh	VVC	-0.487	0.022	1.000	0.436
rh	TE2p	-0.051	0.858	1.000	0.961
rh	V3CD	-0.128	0.590	1.000	0.807
rh	LO1	0.026	0.952	1.000	0.973
rh	LO2	-0.128	0.590	1.000	0.807
rh	LO3	-0.231	0.306	1.000	0.765
rh	V4t	0.231	0.306	1.000	0.765
rh	MT	-0.103	0.675	1.000	0.860
rh	MST	-0.154	0.510	1.000	0.765
rh	FST	0.154	0.510	1.000	0.765
rh	PH	0.205	0.367	1.000	0.765
rh	V3B	-0.231	0.306	1.000	0.765
rh	IP0	-0.077	0.765	1.000	0.936
rh	MIP	-0.051	0.858	1.000	0.961
rh	LIPv	-0.179	0.435	1.000	0.765
rh	LIPd	-0.256	0.252	1.000	0.765
rh	AIP	0.128	0.590	1.000	0.807
rh	PGp	-0.231	0.306	1.000	0.765

rh	TPOJ3	-0.385	0.076	1.000	0.546
rh	TPOJ2	-0.154	0.510	1.000	0.765
rh	TPOJ1	-0.256	0.252	1.000	0.765
rh	STV	0.179	0.435	1.000	0.765
rh	6mp	0.231	0.306	1.000	0.765
rh	6ma	-0.205	0.367	1.000	0.765
rh	SFL	0.051	0.858	1.000	0.961
rh	SCEF	0.000	1.000	1.000	1.000
rh	8BM	0.282	0.204	1.000	0.765
rh	24dd	0.154	0.510	1.000	0.765
rh	24dv	0.103	0.675	1.000	0.860
rh	p32pr	0.179	0.435	1.000	0.765
rh	a32pr	0.436	0.042	1.000	0.436
rh	p24pr	0.026	0.952	1.000	0.973
rh	a24pr	0.205	0.367	1.000	0.765
rh	FEF	-0.205	0.367	1.000	0.765
rh	55b	0.103	0.675	1.000	0.860
rh	PEF	-0.051	0.858	1.000	0.961
rh	6v	0.282	0.204	1.000	0.765
rh	6r	0.154	0.510	1.000	0.765
rh	FOP4	0.410	0.057	1.000	0.484
rh	FOP5	0.308	0.163	1.000	0.724
rh	AVI	0.564	0.007	0.621	0.436
rh	IFSp	-0.256	0.252	1.000	0.765
rh	p9_46v	0.154	0.510	1.000	0.765

Table R5: **Multiple comparison correction of Kendall τ values between duration preference and PSE across ROIs.**

None of the correlations survive these corrections. However, this approach may be overly conservative because the dependencies among our tests are not straightforward to define. For example, ROIs were grouped a priori into functional streams, making it reasonable to apply corrections within each stream rather than across all ROIs. When correcting only within ROIs of the anterior insula, the AVI region in the right hemisphere remains significant, as shown in table R6.

Hemi	ROI	tau	p	p_bonferroni	p_fdr
lh	FOP4	0.333	0.129	0.773	0.155
lh	FOP5	0.436	0.042	0.253	0.084
lh	AVI	0.487	0.022	0.129	0.065
rh	FOP4	0.410	0.057	0.344	0.086
rh	FOP5	0.308	0.163	0.981	0.163
rh	AVI	0.564	0.007	0.040	0.040

Table R6: **Multiple comparison correction of Kendall τ values between duration preference and PSE across ROIs belonging to the anterior insula (AI).**

This highlights the difficulty of defining an appropriate multiple-comparison correction in our case. After careful consideration, we believe that the robustness of Kendall's correlation, combined with a cautious interpretation of the results, mitigates the absence of a formal multiple-comparison correction. We now explicitly note in the manuscript that multiple-comparison correction was not applied (lines 394-395).

Reviewer #2

This study investigates the functional imaging topography of preferred durations along the cortical hierarchy during an interval categorization task. The results showed a large representation of long durations in visual cortex, whereas there is a bias for shorter duration preferences in motor and somatosensory cortices. In addition, all presented durations were topographically organized as interval tuned clusters in parietal and premotor cortices, as well as in the caudal supplementary motor area (SMA). Notably, the rostral SMA, inferior frontal cortex, and anterior insula showed neuronal preferences centered around the mean duration, which correlated with the subjective boundary duration participants employed in

the task. These findings support the notion of a hierarchical organization of duration tuning spanning from sensory to perceptual interval processing. This is a well performed and well analyzed and written study providing novel information regarding the hierarchical organization of cortical duration tuning. Crucially, the authors found distinct functional stages of duration processing, which were implemented in specific cortical regions, that were organized hierarchically, and that supported different functions. Nevertheless, I have a series of main concerns.

1) First, the authors used a categorization task with a memorized bisection point not a discrimination task. Please correct. This is important because there is a large literature on the psychophysics of timing with versions of the categorization task that used training of the prototypes, or as in this case, using training of the bisection point.

We agree with the reviewer that our task does not correspond to a standard discrimination task. We referred to it as a discrimination task as the experiment was originally designed as a discrimination paradigm and later adapted for use inside the MRI scanner by removing the presentation of the reference at each trial. Moreover, the instructions given to participants asked them to report whether the presented stimulus or the memorized stimulus was longer, which is subtly different from explicitly categorizing the current stimulus as shorter or longer. However, we agree that adopting a common frame of reference is necessary, and that describing the task as a categorization task is more appropriate here. We have revised the manuscript to replace all references to the discrimination task with categorization task.

2) Second, since the anterior and posterior sections of SMA have different properties on their topography of preferred durations, I suggest to explicitly dividing these two portions across all the figures and analysis, which will make the story clearer. In fact, there is also a bast literature using the subdivision of SMAproper and preSMA, especially in non-human primates.

We understand the reviewer's point and agree that there is extensive evidence for a functional segregation within the SMA into pre-SMA and SMA proper. However, we would like to outline several reasons why, in our view, imposing this subdivision a priori may not be appropriate in our case. First, to define our regions of interest, we used the Human Connectome Project atlas[9]. According to this parcellation, the subdivision of SMA into 6ma, 6mp, SFL, and SCEF provides a more detailed anatomical and functional characterization than the classical division into pre-SMA and SMA proper[10]. In our analysis, we further grouped these core SMA regions with areas bordering the medial superior frontal gyrus (8BM) and the anterior cingulate cortex (24dd, 24dv, p32pr, a32pr, p24pr, a24pr), as they were all associated with the same functional cluster (see *Methods - Regions of interest (ROIs) identification*). Figure R4 illustrates these ROIs on an inflated average brain, color-coded according to the posterior–anterior segregation of tuning properties that we observed.

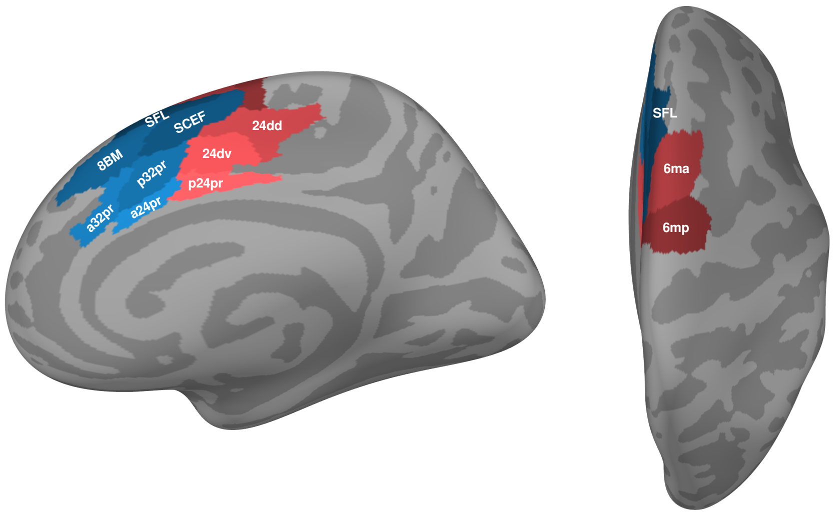

Figure R4: **SMA regions.** Regions from the Human Connectome Project atlas classified as SMA are shown on the right hemisphere of an inflated average brain. Colors indicate the anterior–posterior segregation of duration tuning properties observed in our analyses, with anterior regions in shades of blue and posterior regions in shades of pink.

Starting from this parcellation, a direct mapping onto the distinction between pre-SMA and SMA proper is not straightforward. Accordingly, we described our findings in terms of a posterior-anterior segregation and related them to the pre-SMA/SMA proper literature only in the Discussion. Second, to our knowledge, differences in duration tuning across human SMA subregions have not been reported previously, and we only noticed this pattern as we worked through the analyses. Therefore, we did not have a strong rationale to impose any segregation from the outset. Finally, subdividing the SMA into pre-SMA and SMA proper would affect only the stream-level analyses, where ROIs are collapsed, and not ROI-level analyses, where ROIs are treated independently. Together, these two levels ensure that any grouping of SMA regions does not hide patterns at a finer spatial resolution. For these reasons, we decided to retain the original grouping, but we have revised all figures to explicitly arrange the posterior and anterior SMA ROIs together, making the observed segregation clearer. The updated figures can be found in the main text (Figures 3, 4, 6, and 7) and supplementary materials (Supplementary Figures S3, S4, and S5). Finally, we have updated the description of the ROIs in the Results section (lines 127-129) and in Table 1 to reflect this new ordering. Please also note that this rearrangement minimally changed the regression coefficients and statistics in Figures 4d and 5d. We hope the reviewer will agree that this choice provides the most faithful and informative representation of the data for the readers.

3) Third, I suggest comparing the categorical behavior of the subjects with the BOLD signal during the SCI. Thus, psychometric decisions can be compared with the choice probability calculated from the betas weights of the model for correct and incorrect decisions for each duration and each subject. This analysis could perfectly complement the PSE analysis on the duration tuning distributions.

We thank the reviewer for suggesting this very interesting analysis. This approach is indeed highly informative and widely used in the electrophysiology field. However, it may be less well suited for fMRI data because of the inherently noisy nature of the BOLD signal. In particular, a robust estimation of single-trial beta weights is difficult to obtain, especially in fast event-related designs with brief events such as the present one. Nevertheless, we attempted this analysis to evaluate its feasibility. We first performed a single-trial GLM using the GLMsingle toolbox[4]. For each trial, we modeled activity using a single boxcar covering the shortest possible SCI. We then focused on beta weights from brain regions where tuning was linked to PSE in our previous analyses. Figure R5 shows beta weights from the AVI region of one example participant.

Panel a shows the beta values for individual trials (% signal change) grouped by the participant's short and long responses. As can be seen, the distributions largely overlap, with no clear separation between response types. In panel b, where beta values are further separated by stimulus duration, it is possible to appreciate the Gaussian pattern we capture with the pRF modeling (i.e., beta weights are on average higher for middle durations), but short and long responses (color-coded) remained largely overlapping. These patterns likely reflect a noisy estimation of single-trial betas, which can be particularly pronounced in frontal areas such as AVI, where BOLD signal is complex and driven by multiple processes. Overall, these results already argue against the feasibility of categorizing short and long responses based on single-trial beta weights. Nevertheless, we tried several univariate and multivariate approaches:

1. Univariate
    (a) Using tuning information as likelihood function to predict "long" responses from individual beta weights
    (b) Performing a ROC analysis to estimate the performance of an ideal observer in discriminating between short and long responses given the available beta weights
2. Multivariate
    (a) Training a classifier to decode short and long responses from the pattern of beta weights of the ROI
    (b) Applying a principal component analysis to search for distinct patterns for short and long responses in the beta weights of the ROI

Unfortunately, all of them yielded poor results. As an example, the outcome of the ROC analysis is shown in Figure R5c. We computed hit rates as the proportion of long response trials associated with a beta value greater than a given criterion, and false alarm rates as the proportion of short response trials greater than the same criterion. We tested criteria spanning the full beta distribution and computed the area under the ROC curve for each duration using trapezoidal numerical integration. We then fitted a sigmoid function to the results of the integration and kept only vertices with >60% variance explained. The average profile across these vertices is shown in Figure R5c in black. We excluded durations of 0.2 and 0.8 s because no incorrect responses occurred at those extremes, preventing the computation of hit and false-alarm rates. As can be seen, the neurometric function does not resemble the participant's psychometric function shown in gray.

Overall, we believe that the poor outcome of these analyses does not undermine our main findings on the link between

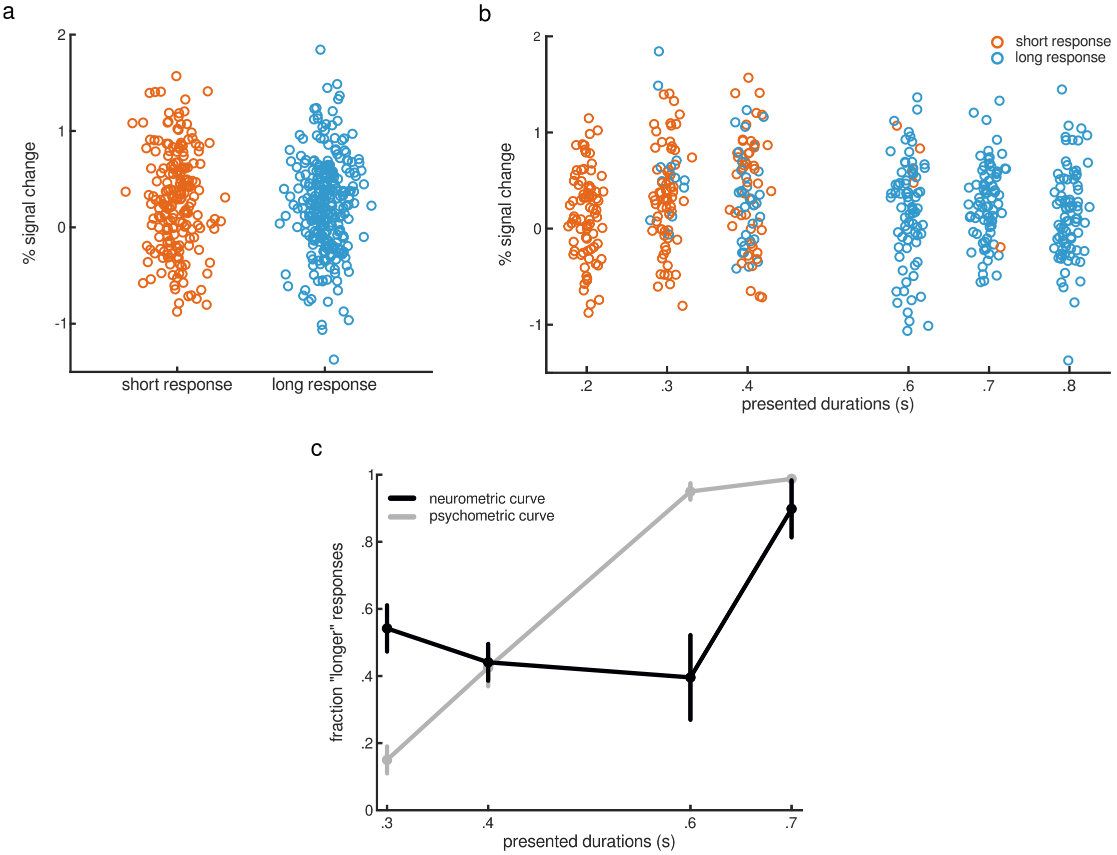

Figure R5: **Neurometric analysis.** **(a)** The plot shows single-trial beta weights from the left AVI region of one example participant. Beta weights, converted to percent signal change, are grouped along the x-axis by the participant's short (orange) and long (light-blue) responses in the duration categorization task. Each dot corresponds to the beta weight from one trial, averaged across vertices within the ROI. **(b)** The same data shown in (a) are plotted grouped along the x-axis by stimulus duration. It is possible to observe that, on average, beta weights are higher for the 0.4 s stimulus duration, consistent with the participant's PSE value of 0.42 s. **(c)** The neurometric curve obtained from the ROC analysis (black) is shown together with the psychometric curve (gray). Black dots correspond to the average ROC profile of vertices with a sigmoid fit explaining more than 60% of variance, and their error bars represent standard deviation. Gray dots correspond to the participant's average performance in the categorization task, and their error bars represent standard error.

duration tuning and the PSE, as single-trial approaches are inherently challenging with fMRI data and need dedicated experimental designs[11]. Nevertheless, we would welcome any feedback on these attempts or additional suggestions from the reviewer.

4) Finally, the duration tuning without a response modulation for spatial location suggests that the reported timing visual areas are high in the visual processing hierarchy. Nevertheless, the fact that they showed a large bias towards long durations may be an indication of sensory adaptation on short durations instead of accumulation. Indeed, ramping activity is usually not reported in primary sensory areas. Please discuss.

We thank the reviewer for raising this interesting point of discussion. First, it is important to clarify that the selection of the reported areas does not depend on the duration tuning model employed. Areas were selected independently of our pRF analysis, using t-value clusters (see Figure 1c) from a GLM that identified locations responding significantly to duration at the group-level (see *Methods - Regions of interest (ROIs) identification*). The absence of clusters in early visual areas is only due to the fact that their activations do not pass the statistical threshold applied at the group-level. This likely reflects the fact that responses are localized to neuronal populations tuned to the spatial position of the stimulus, resulting, in early visual cortex, in small, spatially-specific activations with high inter-subject variability, both anatomically and functionally. Consequently, they do not survive the stringent group-level statistical threshold, which instead favors large and consistent activations across participants. The duration tuning model is then evaluated only within those "GLM-significant" regions. Importantly, this model is specifically designed to capture modulations of brain

activity driven by stimulus duration while ignoring those driven by spatial location and location–duration interactions. Nevertheless, the reviewer is right to point to sensory adaptation as a potential confound in visual timing areas, and this was also one of our major concerns when designing the study. For this reason, we used a stimulus optimized to minimize any form of low-level sensory adaptation (see *Methods - Stimuli and Procedure - Stimuli*). In short, the stimulus consisted of a colored patch whose pixel values changed in each frame following a Gaussian distribution centered on the luminance and color of the background. In this way, local low-level stimulus properties (e.g., luminance, contrast, pixel correlation patterns) changed continuously in each frame, preventing sensory adaptation, while globally they remained constant and independent of stimulus duration (e.g., mean luminance and color matched the background). In addition, we presented the stimuli sequentially across the different spatial positions and with long inter-trial intervals (between 1.8 and 2.5 s). On average, the stimulus appeared in the same spatial location approximately every 20 s, allowing the BOLD signal to return to baseline and minimizing repetition suppression. It is also important to mention that a growing body of studies has reported monotonic/ramping activity in early visual and somatosensory regions in response to stimulus duration, both in electrophysiological recordings[12], [13] and in human fMRI[6], [8], [14]. Importantly, ramping activity in early visual areas has been reported also for empty stimuli[5] and for time of expected reward[15], where sensory input is minimal or absent. Finally, in our previous study[7] we directly compared the cross-validated goodness of fit of Gaussian and monotonic models of duration tuning along the dorsal visual stream and found, similarly to [6], a transition between these two types of responses. While the monotonic model better explains activity in early visual areas, the Gaussian one wins in parietal and frontal regions. If sensory adaptation were actually driving the monotonic pattern in early visual areas, the monotonic model would not be able to explain more variance than the Gaussian one. Indeed, the Gaussian model would better capture a response that is suppressed for short durations and peaks only for long ones. Figure R6 (included as supplementary information in [7]) depicts the activity of one example vertex best fitted by the monotonic model.

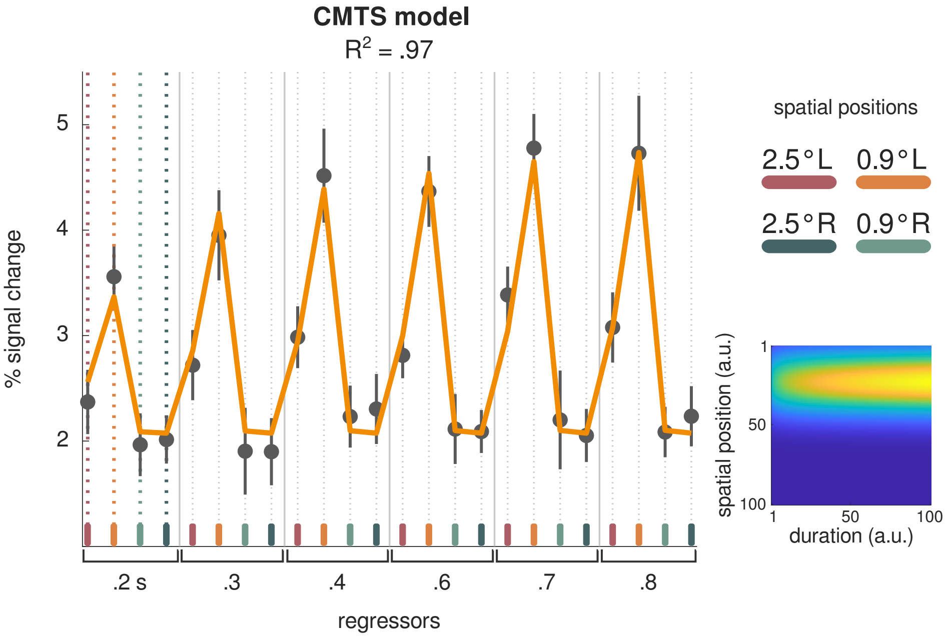

Figure R6: **Compressive monotonic model of duration tuning.** The plot shows the fit in one example vertex of the Compressive Monotonic Time and Gaussian Space (CMTS) model, previously developed and tested in [7]. Dots represent the GLM β estimates, converted to percent signal change, for each combination of stimulus duration and spatial location in our experimental manipulation. Stimulus locations (color-coded) and durations are grouped along the x-axis. The solid line represents the model fit. The model is described by the following formula: $\textit{neuronal response} \sim (m \cdot d)^c \cdot e^{-\frac{(s-\mu_s)^2}{2\sigma_s^2}}$, where μ_s and σ_s define the vertex's spatial (s) receptive field, and m and $c \leq 1$ describe the modulation driven by stimulus duration (d), in terms of slope and compression, respectively. The overall receptive field profile is shown in the inset at the lower right. Modified from [7].

The black dots and corresponding error bars represent the percent signal change associated with the different combinations of stimulus position and duration. Activity peaks for a preferred spatial location and gradually scales with stimulus

duration. However, the increase is modest (see also Figure 6a in [5] for a quantification) and accompanied by largely overlapping standard errors, suggesting that it is unlikely to be driven by sensory adaptation. In summary, we are confident that the observed results cannot be attributed to simple sensory adaptation. We agree with the reviewer, however, that the functional role of monotonic responses in duration processing and perception remains to be clarified and further explored. We are actively investigating this question (see also response to reviewer 1, point 4) through a series of studies, some of which are currently in preparation (e.g., [16]).

We have revised the Discussion section (lines 435-439) to better frame our interpretation of the monotonic tuning within the observed results and the existing literature. The paragraph now reads as follows:

The first processing stage may occur in ventral and lateral visual areas, where we consistently observed a preference for long durations. This type of preference is compatible with a ramping response to stimulus duration, in line with previous findings showing that neuronal activity in early visual regions increases modestly yet monotonically as a function of stimulus duration[5]–[7], [12].

Minor comments:

5) Please use large fonts in the figure.

We thank the reviewer for this suggestion which has helped us improve the quality of data visualization in the manuscript. We have now modified several aspects of both the main and supplementary figures, including font sizes wherever possible, contrasts, and the visibility of axes and individual data points. Because the final figure dimensions depend on the journal's layout, we will work with the Editorial Office to ensure that all fonts are appropriately scaled in the final version, should our manuscript be accepted.

6) Please consider the change of contrast of the ROIS with respect to the inflated brain of Figure 1C, it is difficult to distinguish their limits.

We appreciate the reviewer's suggestion and have now increased the visibility of the ROIs' borders in Figure 1c. Because the ROI contrasts remained constrained by the need to display the underlying t-statistics clusters, we have included an additional supplementary figure (S1) showing the clusters and ROIs in two separate panels, allowing for clearer recognition of both.

7) I strongly suggest using graph analysis on the matrix data of Figure 6.

We thank the reviewer for this suggestion, which nicely complements our analysis. We have included a supplementary figure (S6) showing the network representation of the correlation matrix at the ROI level. In this figure, edge color and width indicate correlation value, and node color denotes Louvain community membership. The Louvain method[17] is a greedy algorithm that finds clusters in a network by iteratively grouping nodes to maximize modularity (Q):

$$Q = \frac{1}{2m} \sum_{i=1}^{N} \sum_{j=1}^{N} \left[A_{ij} - \frac{k_i k_j}{2m} \right] \delta(c_i, c_j), \quad (2)$$

where A_{ij} is the adjacency matrix; k_i and k_j are the sum of weights of node i and j; m is the total sum of weights; N is the total number of nodes; c_i and c_j represent the clusters to which i and j belong; δ is a delta function equal to 1 if $c_i = c_j$ and 0 otherwise. In brief, Q measures how strongly nodes are connected within each cluster relative to nodes outside. Consistent with what we reported in the manuscript, the Louvain community detection algorithm revealed the presence of three clusters that largely matched the structure shown in the dendrogram in Figure 6. In addition, we computed for each node several metrics of centrality, hubness and authority as well as degree and weighted degree distributions. These results will be made available as Gephi and Gephi Lite[18] GEXF and JSON files, allowing interested readers to explore the network, visualize the results and run additional analyses directly from their web browser. However, we would like to point out that the correlation matrix yields a fully connected graph with both positive and negative weights. For this reason, the results of some standard graph and network analyses (including those of the Louvain algorithm) require cautious interpretation. This is why we decided not to include this analysis in the main manuscript but to report it as supplementary information. Finally, we would like to emphasize that the dendrogram presented in Figure 6 is the outcome of a hierarchical clustering analysis performed on the correlation matrix at the stream level using the most conservative linkage method (see *Methods - Analysis of long-range relationships between duration preferences*) and therefore provides a proper quantification of the structure of the correlation matrix.

We refer to this new network analysis in the Methods section (lines 898-905) as follows:

We investigated whether and how changes in duration preferences are related along the cortical hierarchy by performing a correlation analysis across areas. For each participant, we computed the median duration preference within each ROI, including both hemispheres for bilateral ROIs. We then calculated the Kendall correlation matrix across areas using the cor function from the R stats package. We treated this matrix as an adjacency matrix (see S6 Fig) and characterized the resulting network by computing, for each node, its degree, centrality, hubness, and authority using Gephi. In addition, we applied the Louvain community detection algorithm to understand the structure of relationships among the ROIs.

8) Please consider referring to the work of Merchant et al., 2024 Adv Exp Med Biol regarding the core timing network.

We thank the reviewer for suggesting this work, which is indeed relevant to our findings and to the discussion of SMA tuning properties. We have now added the reference in line 556 of the Discussion.

9) It would be great to run subjects in an experiment with two blocks of intervals to determine how the range of intervals and the position of the bisection point modulate their functional tuning distributions, their topographic organization, and their relationship with the psychometric functions (PSE and choice probability). I know that this implies running a new experiment, but it would be nice to at least discuss the advantages of this experimental design.

We fully agree with the reviewer that such an experimental design would be highly informative for investigating the link between duration tuning and perception. In particular, testing multiple sets of intervals would help (i) assess whether durations are represented in a relative or absolute manner, and (ii) better characterize duration tuning of "boundary" populations. Regarding the first point, a pivotal study on Gaussian duration tuning in humans[19] showed that temporal maps in SMA adaptively reshaped when a wider duration range was tested, with a small proportion of vertices preserving the same selectivity across ranges. This suggests that duration may be represented in a relative fashion, at least in SMA, although targeted studies are still necessary to characterize the flexibility of duration tuning and topography across the cortical hierarchy. Regarding the second point, the first study reporting neuronal activity linked to the PSE in monkey medial premotor cortex employed three different duration ranges in a categorization task[2]. When the categorization boundary changed, the peak activity of a subset of boundary neurons shifted accordingly, reflecting the monkeys' PSE in the new range, as also mentioned in our manuscript (lines 505-506). Together, these findings indicate that testing multiple temporal ranges has strong potential to advance our understanding of the mechanisms addressed in the present study. Such a design would also allow the bisection point to be set independently of the mathematical mean of the range, helping to disentangle the respective roles of stimulus statistics and task demands in shaping frontal activity for temporal processing. We are currently running an experiment in this direction[3]. To acknowledge these points in the manuscript, we have added the following sentence to the Discussion (lines 597-601):

Finally, a proper characterization of "boundary" neuronal populations would benefit from an experimental design with multiple duration ranges[2], which would allow assessing whether their activity shifts, as the PSE does, according to different reference durations in the task. Such a design could also help to better establish the properties and functional role of duration tuning and its topographic organization across the other stages of duration processing.

References

[1] A. Lage-Castellanos, G. Valente, M. Senden, and F. De Martino, "Investigating the Reliability of Population Receptive Field Size Estimates Using fMRI," *Front. Neurosci.*, vol. 14, p. 541 186, Jul. 2020, ISSN: 1662-453X. DOI: 10.3389/fnins.2020.00825.

[2] G. Mendoza, J. C. Méndez, O. Pérez, L. Prado, and H. Merchant, "Neural basis for categorical boundaries in the primate pre-SMA during relative categorization of time intervals," *Nat. Commun.*, vol. 9, no. 1098, pp. 1–17, Mar. 2018, ISSN: 2041-1723. DOI: 10.1038/s41467-018-03482-8.

[3] V. Centanino, G. Fortunato, and D. Bueti, "How ensemble temporal statistics influence duration perception of visual events," *J. Vis.*, vol. 25, no. 9, p. 2496, Jul. 2025, ISSN: 1534-7362. DOI: 10.1167/jov.25.9.2496.

[4] J. S. Prince, I. Charest, J. W. Kurzawski, J. A. Pyles, M. J. Tarr, and K. N. Kay, "Improving the accuracy of single-trial fMRI response estimates using GLMsingle," *eLife*, Nov. 2022. DOI: 10.7554/eLife.77599.

[5] J. Zhou, N. C. Benson, K. N. Kay, and J. Winawer, "Compressive temporal summation in human visual cortex," *Journal of Neuroscience*, vol. 38, no. 3, pp. 691–709, 2018, ISSN: 0270-6474. DOI: 10.1523/JNEUROSCI.1724-17.2017.

[6] E. Hendrikx, J. M. Paul, M. van Ackooij, N. van der Stoep, and B. M. Harvey, "Visual timing-tuned responses in human association cortices and response dynamics in early visual cortex," *Nat. Commun.*, vol. 13, no. 3952, pp. 1–19, Jul. 2022, ISSN: 2041-1723. DOI: 10.1038/s41467-022-31675-9.

[7] V. Centanino, G. Fortunato, and D. Bueti, "The neural link between stimulus duration and spatial location in the human visual hierarchy," *Nat. Commun.*, vol. 15, no. 10720, pp. 1–19, Dec. 2024, ISSN: 2041-1723. DOI: 10.1038/s41467-024-54336-5.

[8] I. Kim, E. R. Kupers, G. Lerma-Usabiaga, and K. Grill-Spector, "Characterizing Spatiotemporal Population Receptive Fields in Human Visual Cortex with fMRI," *J. Neurosci.*, vol. 44, no. 2, Jan. 2024, ISSN: 0270-6474. DOI: 10.1523/JNEUROSCI.0803-23.2023.

[9] M. F. Glasser, T. S. Coalson, E. C. Robinson, C. D. Hacker, J. Harwell, E. Yacoub, K. Ugurbil, J. Andersson, C. F. Beckmann, M. Jenkinson, *et al.*, "A multi-modal parcellation of human cerebral cortex," *Nature*, vol. 536, no. 7615, pp. 171–178, Aug. 2016, ISSN: 1476-4687. DOI: 10.1038/nature18933.

[10] C. M. Baker, J. D. Burks, R. G. Briggs, J. R. Sheets, A. K. Conner, C. A. Glenn, G. Sali, T. M. McCoy, J. D. Battiste, D. L. O'Donoghue, *et al.*, "A Connectomic Atlas of the Human Cerebrum—Chapter 3: The Motor, Premotor, and Sensory Cortices," *Operative Neurosurgery*, vol. 15, no. Suppl 1, S75, Sep. 2018. DOI: 10.1093/ons/opy256.

[11] H. Abdulrahman and R. N. Henson, "Effect of trial-to-trial variability on optimal event-related fMRI design: Implications for Beta-series correlation and multi-voxel pattern analysis," *Neuroimage*, vol. 125, pp. 756–766, Jan. 2016, ISSN: 1053-8119. DOI: 10.1016/j.neuroimage.2015.11.009.

[12] J. Duysens, S. J. Schaafsma, and G. A. Orban, "Cortical off response tuning for stimulus duration," *Vision Res.*, vol. 36, no. 20, pp. 3243–3251, Oct. 1996, ISSN: 0042-6989. DOI: 10.1016/0042-6989(96)00040-5.

[13] S. Reinartz, A. Fassihi, M. Ravera, L. Paz, F. Pulecchi, M. Gigante, and M. E. Diamond, "Direct contribution of the sensory cortex to the judgment of stimulus duration," *Nat. Commun.*, vol. 15, no. 1712, pp. 1–15, Feb. 2024, ISSN: 2041-1723. DOI: 10.1038/s41467-024-45970-0.

[14] A. Stigliani, B. Jeska, and K. Grill-Spector, "Encoding model of temporal processing in human visual cortex," *Proc. Natl. Acad. Sci. U.S.A.*, vol. 114, no. 51, E11047–E11056, Dec. 2017. DOI: 10.1073/pnas.1704877114.

[15] M. G. Shuler and M. F. Bear, "Reward timing in the primary visual cortex," *Science*, vol. 311, no. 5767, pp. 1606–1609, Mar. 2006, ISSN: 1095-9203. DOI: 10.1126/science.1123513.

[16] D. Bueti, A. Toso, G. Fortunato, C. Zanonato, and M. Diamond, "A distinct functional role of primary visual and medial premotor cortex in visual duration processing," *J. Vis.*, vol. 25, no. 9, p. 2636, Jul. 2025, ISSN: 1534-7362. DOI: 10.1167/jov.25.9.2636.

[17] V. D. Blondel, J.-L. Guillaume, R. Lambiotte, and E. Lefebvre, "Fast unfolding of communities in large networks," *J. Stat. Mech.: Theory Exp.*, vol. 2008, no. 10, P10008, Oct. 2008, ISSN: 1742-5468. DOI: 10.1088/1742-5468/2008/10/P10008.

[18] M. Bastian, S. Heymann, and M. Jacomy, "Gephi: An Open Source Software for Exploring and Manipulating Networks," *ICWSM*, vol. 3, no. 1, pp. 361–362, Mar. 2009, ISSN: 2334-0770. DOI: 10.1609/icwsm.v3i1.13937.

[19] F. Protopapa, M. J. Hayashi, S. Kulashekhar, W. van der Zwaag, G. Battistella, M. M. Murray, R. Kanai, and D. Bueti, "Chronotopic maps in human supplementary motor area," *PLOS Biology*, vol. 17, no. 3, pp. 1–34, Mar. 2019. DOI: 10.1371/journal.pbio.3000026.
